# Rapid, Multispecies Detection of SARS-CoV-2 Antibodies via a Meta-Surface Plasmon Resonance Biosensor

**DOI:** 10.1155/2024/9350822

**Published:** 2024-01-19

**Authors:** Ya Zhao, Rui Li, Zuqing Liu, Hanlin Zhou, Jingyu Yang, Shaoran Zhang, Liping Huang, Gang L Liu, Qiang Zhang, Meilin Jin

**Affiliations:** ^1^College of Veterinary Medicine, Huazhong Agricultural University, Wuhan 430070, China; ^2^National Key Laboratory of Agricultural Microbiology, Huazhong Agricultural University, Wuhan 430070, China; ^3^College of Life Science and Technology, Huazhong University of Science and Technology, 1037 Luo Yu Road, Wuhan 430074, China; ^4^Liangzhun (Shanghai) Industrial Co. Ltd., 1582 Gu Mei Road, Shanghai 200233, China; ^5^Hubei Jiangxia Laboratory, Wuhan 430200, China; ^6^College of Biomedicine and Health, Huazhong Agricultural University, Wuhan 430070, China

## Abstract

Public health concerns have been raised by numerous reports of severe acute respiratory syndrome coronavirus type 2 (SARS-CoV-2) and its variations infecting a range of animals. Wildlife reservoirs may facilitate the evolution of viral types capable of causing human infection in the future. Therefore, epidemiological monitoring of animals in close contact with humans is necessary. Yet, infection symptoms are not obvious in most animals, which leads to a short nucleic acid test-detection period and limits the application of this method in animals. The use of virus- and pseudovirus-based neutralizing antibody detection techniques is restricted to establishments with elevated biosafety standards. Traditional enzyme-linked immunosorbent assays (ELISA) do not offer multispecies detection and are time-consuming and labor-intensive. This work developed a polyethyleneimine-gold nanoparticle meta-surface plasmon resonance biosensor system-based multispecies SARS-CoV-2 antibody detection platform that is fast, sensitive, has a high throughput, and is fully automated. The test can be done in 30 min and specificity is up to 100% for detection in cats, dogs, and minks. Moreover, the coincidence rate was up to 99.36% (313/315) for the detection of pseudovirus in clinical and immune sera. Additionally, this method's detection sensitivity in cat, dog, and mink serum is 2,048, 1,024, and 4,096 times, which is much better than indirect ELISA and comparable to indirect immunofluorescence assays. An efficient method for COVID-19 epidemiology screening in animal serum will be made available by this platform.

## 1. Introduction

In December 2019, a pneumonia outbreak with an unclear etiology occurred in Wuhan, China, which gave rise to COVID-19. The cause was later identified as severe acute respiratory syndrome coronavirus type 2 (SARS-CoV-2). To date, SARS-CoV-2 has resulted in over 772 million confirmed cases and over 6.98 million confirmed deaths. SARS-CoV-2 has quickly changed into new dominant varieties, including Beta, Delta, and Omicron, which have spread widely around the world even though mass vaccination has successfully helped control the epidemic and restrict morbidity and mortality [1, 2]. Breakout infections and decreases in the effectiveness of neutralizing antibodies (NAs) caused by variants were widely reported [3, 4]. As a result, this epidemic presents a significant risk to both economic growth and public health worldwide [5, 6].

Research has revealed that the cellular entrance receptor angiotensin I converting enzyme 2 (ACE2) is shared by the spike proteins of SARS-CoV and SARS-CoV-2. Thus, the spike protein became the preferred target for the design of SARS-CoV-2 vaccines and some diagnostic products. It also suggests that SARS-CoV-2 may have the same or wider host range as SARS-CoV [6–8]. While the identity of SARS-CoV-2's intermediate host is yet unknown, numerous infections, as well as serological and receptor affinity tests for spike protein, have been verified in a variety of wildlife species, including chimpanzees, rhesus and crab-eating monkeys, hamsters, ferrets, minks, rabbits, white-tailed deer, and civets. Companion animals with close contact with humans, like dogs and cats, can also be infected [9–17]. Furthermore, once SARS-CoV-2 is introduced into animal hosts, it is extremely vulnerable to alterations that promote cross-species transmission [18]. One study reported that the Omicron variant probably originated in mice, evolved in mouse hosts, and transmitted to humans. Meanwhile, another study reported that SARS-CoV-2 could infect white-tailed deer, spread and mutate more widely deer-to-deer, and spread back to humans [19]. One of the biggest worries is the mink variety, which can spread to humans and cause additional community transmission. Consequently, many minks were culled to prevent the spread of the mutated virus [20–22]. Most importantly, SARS-CoV-2-infected animals do not display obvious clinical symptoms, and infections would be undetected without routine diagnosis [23], which raises concerns for public health and agricultural economic losses. Therefore, in vulnerable animals, long-term surveillance of the SARS-CoV-2 virus and antibody variations is required.

Research has revealed that the virus does not affect all animals in the same way. However, virus detection using RT-PCR is only possible for animals for a short time, which greatly increases the chances of false and missed tests [14, 24]. Among different animal serological detection methods, NAs from live or pseudotyped viruses are the gold standard. However, biosafety level 2 or 3 (BSL-2 or BSL-3) facilities are required, which has hindered the widespread use of this method [25, 26]. The assay is based on the competitive suppression of the SARS-CoV-2 spike receptor-binding domain (RBD)-ACE2 receptor interaction. Although not limited by species, this method can only detect competitive NAs, which may be missed in some individuals that contain only binding antibodies [27, 28]. Traditional enzyme-linked immunosorbent assays (ELISAs) are time-consuming and labor-intensive, and the horseradish peroxidase-conjunction anti-antibody is difficult to prepare for the corresponding animal [29]. Because they are easy to use and identify SARS-CoV-2 antibodies quickly, lateral flow immunoassays and chemiluminescence are better point-of-care methods. However, these assays are mainly developed targeting human rather than animal serum [30, 31]. Therefore, the development of an effective, highly sensitive, rapid, and accurate multispecies diagnosis of SARS-CoV-2 would help efficiently evaluate spillover infection and prevalence of SARS-CoV-2 in different animal populations and determine whether a COVID-19 vaccine is needed in susceptible animals. This approach could potentially evaluate the duration and efficacy of vaccinations for a population who has already received them, assisting in the decision of whether a COVID-19 vaccine booster is required.

In response to this pressing need, we have created a novel biosensor platform using meta-surface plasmon resonance (MetaSPR) for the quick, accurate, and quantitative detection of SARS-CoV-2 antibodies in a variety of animals, not just cats, dogs, and minks. Our MetaSPR biosensor platform can achieve high-throughput detection of antibodies from SARS-CoV-2 without signal amplification or multiple washing procedures within 30 min [32, 33]. In addition, the MetaSPR biosensor chips were integrated within 96 microwell plates so the measurements could be carried out in generic microplate readers, further enhancing its high-throughput, portable, and point-of-care capabilities.

## 2. Materials and Methods

### 2.1. Gene Cloning, Protein Expression, Purification, and Virus Culture and Inactivation

The plasmid-expressing SARS-CoV-2 spike (S)-trimer protein (pCDNA3.1-S-trimer-his) was constructed in our laboratory and transfected into HEK293 cells following the manufacturer's recommendations (Sino biological, Beijing, China) for production of recombinant proteins. On Day 4 after transfection, the supernatant was filtered and collected, and the protein was affinity-purified on a 5 ml his-tagged resin (Ni Sepharose™ excel, Cytiva, USA), followed by size-exclusion chromatography on a Superdex 200 increase column (Cytiva, USA) equilibrated in phosphate-buffered saline (PBS). As in our previous study [34], SARS-CoV-2 strain Wuhan-Hu-1 (NC_045512) was propagated in Vero-E6 cells (ATCC® CRL-1586™) with Dulbecco's modified Eagle medium (DMEM; Hyclone, Logan, UT, USA) supplemented with 2% (v/v) fetal bovine serum (PAN Biotech, Germany), 100 U/ml penicillin-streptomycin (Thermo Fisher Scientific, USA) incubated at 37°C in 5% CO_2_. Finally, the virus was completely inactivated with *β* -propiolactone. Work with live viruses was performed in BSL-3 facilities at Huazhong Agricultural University.

### 2.2. Clinical Sera

Negative sera from dogs (*n* = 51), cats (*n* = 53), rabbits (*n* = 6), and minks (*n* = 52) were obtained before the SARS-CoV-2 epidemic outbreak. Additionally, we confirmed 52 cat and 10 dog-positive samples via sera pseudovirus neutralization tests (pVNT). Three sera were positive for feline infectious peritonitis virus and three more for canine coronavirus.

### 2.3. Immune Sera

We collected immune sera from all animals, including dogs (*n* = 26), cats (*n* = 31), rabbits (*n* = 6), and minks (*n* = 22, 3−5 months of age), which were injected subcutaneously with 30 *μ*g of recombinant spike trimer and inactivated vaccine. Aluminum hydroxide was used as an immune adjuvant. Control groups were injected with aluminum hydroxide and PBS. Immune sera were collected at 7 days after each vaccination or boost. The antibody titer of immune sera was determined by a SARS-CoV-2 pVNT. All of the sera were stored at −80°C before use.

### 2.4. Preparation of GNPS and GNP-Labeled SARS-CoV-2 S-Trimer Protein

In this study, the synthesis of gold particles and the labeling of SARS-CoV-2 S-trimer protein are as follows: First, 1.5 ml of GNPs solution was adjusted to pH 7.4 with 0.1 M K_2_CO_3_ solution, and then 4.9 *μ*l of PBS solution 4.9 *μ*l 0.93 mg/ml SARS-CoV-2 S-trimer protein was added to the colloidal GNP solution and incubated for 15 min. After 15 min of blocking with 22.5 *μ*l of blocking solution (10 mM PBS) containing PEG 20,000 (10%, w/v), the colloidal GNP suspension was centrifuged at 8,000 rpm for 18 min. The precipitate of GNP-labeled S-trimer protein was resuspended in 470 *μ*l of stable buffer (20 mM Tris (pH 9.2), 0.3% sucrose, and 0.05% PEG 20,000) and stored at 4°C until use.

### 2.5. Size-Exclusion Chromatography-High Performance Liquid Chromatography (SEC-HPLC)

The purity of the S-trimer was analyzed by SEC-HPLC using a 1260 Infinity HPLC system (Agilent, USA) with an analytic TSK gel G3000SWxL column (Tosoh, Japan). PBS was used as the mobile phase with OD_280_ nm detection over a 60 min period at a flow rate of 0.5 ml/min.

### 2.6. Biolayer Interferometry (BLI) Binding Assay

The kinetics of human ACE2-HFc binding to S-trimer were measured by a BLI binding assay on a FortéBio Octet RED96e system, using Protein A biosensors and following the manufacturer's instructions. The assay followed sequential steps at 27°C as follows: (1) Baseline: biosensors were immersed in PBS with 0.02% (v/v) Tween (PBST) for 60 s. (2) Loading: biosensors were immersed in 50 *μ*g/ml with ACE2-HFc for 120 s. (3) Baseline 2: biosensors were immersed in PBST for 60 s to reach the baseline. (4) Association: biosensors were immersed in a three-fold serially diluted S-trimer for 180 s. (5) Dissociation: biosensors were immersed in 0.02% (v/v) PBST for 180 s to reach the baseline. The value of buffer control binding was subtracted to deduct nonspecific binding. Kon, Koff, and Kd were calculated with FortéBio Data Analysis software (Octet Analysis Studio 12.2), using 1 : 1 binding and a global fitting model.

### 2.7. Affinity Measurement Using the MetaSPR Biosensor

Before conducting the affinity assay, protein A-modified MetaSPR chip wells were added 20 *μ*g/ml with ACE2-HFc for 300 s. Then, 50 *µ*l of a series of concentrations of S-trimer (0–100 nM), both in two-fold serial dilutions in PBST buffer, were simultaneously added into the MetaSPR chip wells. The association period was measured with respect to time for 5 min using the WeSPR200 instrument. After the association was completed, 150 *µ*l of PBST buffer was added to the same chip wells, and the dissociation dynamic curves were immediately monitored. The PBST buffer was used as a control to deduct nonspecific binding. Finally, the 1 : 1 binding analysis was performed to estimate the dissociation constants (KD) of the S-trimer interacting with ACE2-HFc using the XLement Data Analysis software (WeSPR200 1.0.2).

### 2.8. The PEI-GNPs MetaSPR Platform Was Used for SARS-CoV-2 Antibody Detection

The MetaSPR sensor detects antibodies to SARS-CoV-2 spike protein in the serum of cats, dogs, rabbits, and minks as follows: first, 100 *μ*l samples (1 : 100 dilution in a solution of PBST containing 2% bovine serum albumin (BSA)) were added to each hole in a pre-Protein A-functionalized sensor chip plate. After 15 min of reaction, the total IgG in the sample was captured on the surface of the sensor chip. The sample was discarded and washed five times with PBST. Then, the OD values of initial spectra at 580 nm wavelength were recorded by a universal microplate reader (WeSPR 200, XLement, China) and 100 *μ*l GNP-labeled SARS-CoV-2 S-trimer protein solution to each well. After 15 min, the final spectral OD value after the reaction was recorded at the wavelength of 605 nm. The relative OD value change is the final spectral OD value minus the initial spectral OD value.

### 2.9. ELISA

A recombinant spike-trimer was used to coat flat-bottom 96-well plates (Thermo Fisher, USA) at a final concentration of 1 *μ*g/ml at 4°C overnight. The next day, plates were washed three times with PBS containing 0.05% (v/v) Tween-20 (PBST). Then, a blocking solution containing 2% (w/v) BSA in PBST was added, followed by a 2-hr incubation at 37°C. After that, serial two-fold dilutions of heat-inactivated serum samples were added and incubated at 37°C for 1 hr. Then, the plates were washed five times with PBST. The horseradish peroxidase-conjugated rabbit-anti-cat, dog (Biodragon, Beijing, China), and anti-mink (Solarbio, Beijing, China) antibodies were diluted 1 : 20,000 in PBST, added to the wells (100 *μ*l/well), and incubated for 30 min at room temperature. After another five washes, 3,3',5,5'-Tetramethylbenzidine substrate (Sigma–Aldrich, USA) was added, and the samples were incubated in the dark at room temperature for 10 min. The reaction was stopped by the addition of 50 *μ*l of stop solution to each well, and the absorbance at 630 nm was measured in a microplate reader (WeSPR 200, XLement, China).

### 2.10. SARS-CoV-2 pVNT Assay

A vesicular stomatitis virus carrying luciferase and SARS-CoV-2 genes was obtained from Vazyme Medical Technology (Nanjing, China). Following the manufacturer's instructions, heat-inactivated serum samples were diluted in duplicate at an initial 1 : 20, followed by a three-fold dilution series. Simultaneously, virus and cell control wells were set up. A sample of 650 TCID_50_-pseudotyped viruses was separately mixed with an equal volume of each diluted serum sample and incubated for 1 hr in a 96-well plate. Next, ACE2-BHK cells were trypsinized, resuspended, and added to the mixture. After 24 hr of culture at 37°C, cells were washed and lysed. Luciferase activity was determined using the Firefly Luciferase Assay system (Promega, USA), and values were determined using a Spark 10 M microplate reader (Tecan, Switzerland). The sample neutralization titers that resulted in 50% inhibition of viral replication (NT_50_) were interpolated from a nonlinear, best-fit curve with GraphPad Prism.

### 2.11. Indirect Immunofluorescence Assay

Vero E6 cells were seeded in 48-well plates and infected with SARS-CoV-2 at a 0.01 multiplicity of infection for 48 hr. Next, cells were fixed with 4% (v/v) paraformaldehyde and permeabilized with 0.1% (v/v) Triton X-100. Then, cells were blocked with 1% (w/v) BSA for 1 hr at 37°C and incubated with the dog and cat SARS-CoV-2-positive sera and mink inactive vaccine immune sera, diluted 1 : 100–1 : 16,000 at 37°C for 2 hr. After three 5-min washes with PBS, cells were incubated with CoraLite488-conjugated rabbit–anticat, dog IgG (Biodragon, Beijing, China), antimink IgG (Solarbio, Beijing, China) at 37°C for 1 hr. After repeating the washing step with PBS, the samples were treated with 4',6-diamidino-2-phenylindole for 10 min for nucleic acid staining. Samples were visualized with the EVOS FL Auto system (Thermo Fisher Scientific, Waltham, MA, USA).

## 3. Results

As previously stated, deficiencies such as low sensitivity and accuracy will arise when dealing with sera or other complicated samples. However, the use of a MetaSPR to detect markers can provide high throughput, rapidity, and reproducibility. Therefore, we propose a high-sensitivity detection platform with PEI-GNPs MetaSPR. Polyethyleneimine (PEI) has high cationic properties and can adsorb anions; thus, it can be adsorbed onto the chip surface in a short time [35, 36]. Similarly, when gold nanoparticles (GNPs) of different particle sizes and plasmonic properties were synthesized by chemical reduction on the chip, PEI could rapidly adsorb them. This method significantly increased the MetaSPR biosensor platform's sensitivity because of the GNPs' potent surface plasmon absorption and excellent light-scattering qualities [37–39].

**Figure 1:**
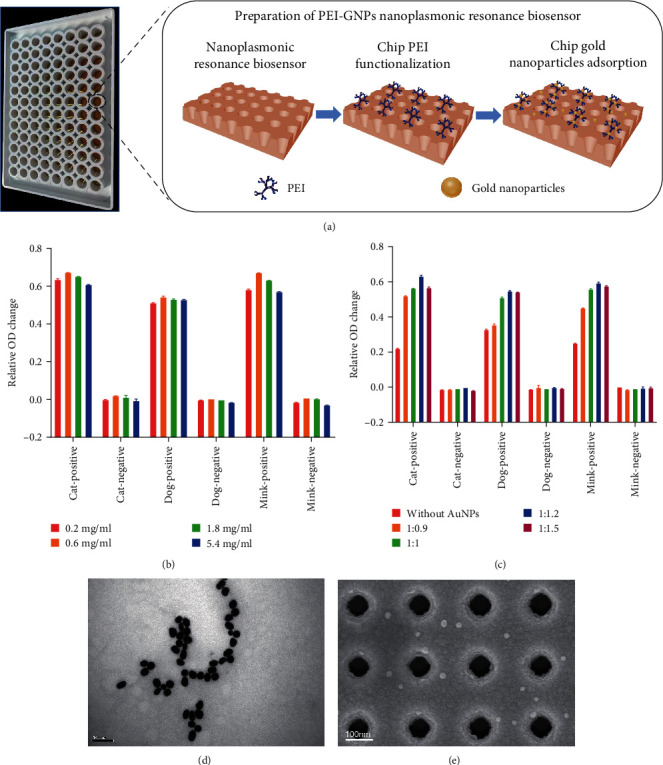
Characterization of the PEI-GNPs MetaSPR biosensor: (a) diagram representation of the mechanism of the MetaSPR biosensor; (b) detection of signal responses in cat, dog, and mink samples using the MetaSPR biosensor set up with different concentrations of PEI; (c) different proportions of PEI and GNP in the MetaSPR biosensor were used to detect signal responses in cat, dog, and mink samples; (d) scanning electron microscopy image of GNPs; (e) scanning electron microscopy image GNPs on a replicated nanocup array.

We uniformly spread the UV-cured polymer onto a mold with a repeating regular arrangement of nanocup structures and topped with a polyethylene terephthalate (PET) sheet. Then, we carefully peeled the PET sheet. Titanium and gold layers (90 nm) were deposited by electron beam evaporation of a coater to form chip substrates. Then, PEI was added to the surface of the chip for 1 hr. Subsequently, we washed twice with DDW. Finally, GNPs were added, the liquid was removed after incubating at room temperature for 1 hr, and the surface of the chip was dried with nitrogen gas. We named the finished chip supersensitive PEI-GNPs MetaSPR biosensor (Figure 1(a)). Detection performance was optimal when 0.6 mg/ml PEI and GNPs were used in a 1 : 1.2 ratio.

Additionally, sera from cats, dogs, and minks had the highest signal-to-noise ratio (Figures 1(b) and 1(c)). In addition, to obtain a more stable and reproducible signal response, GNPs with an average particle size of 30 nm were prepared (Figures 1(d) and 1(e)). GNPs showed good homogeneity and stability.

### 3.1. Expression, Purification, and Characterization of Highly Active S-Trimer Protein

**Figure 2:**
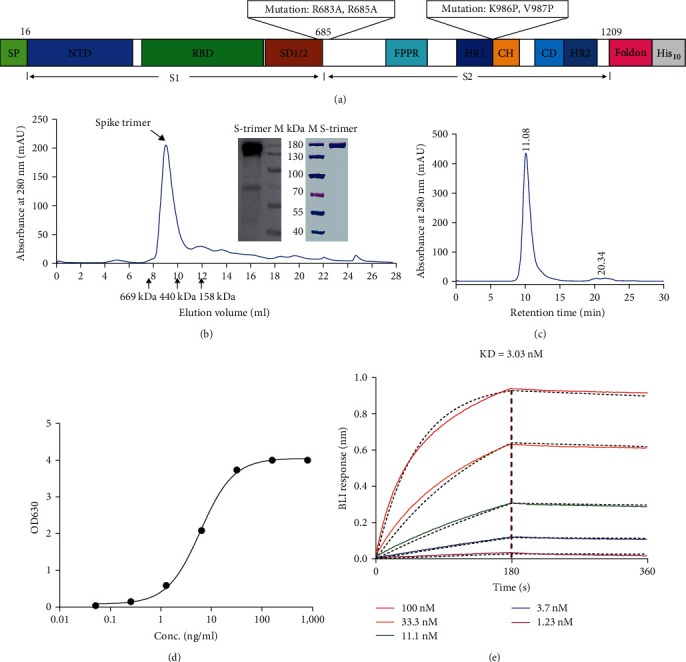
Characterization of the SARS-CoV-2 S-trimer and diagrams of the MetaSPR biosensor assay for S-trimer IgG detection: (a) schematic model of the S-trimer protein; (b) representative elution chromatograph of the recombinant S-trimer protein on size-exclusion chromatography. The figures show reducing SDS–PAGE and western blotting analyses of S-trimer; (c) SEC-HPLC analysis of S-trimer purity; (d) determination of the biological activity (EC_50_) between S-trimer and human ACE2-HFc receptor by ELISA; (e) determination of the binding affinity between S-trimer and human ACE2-HFc receptor by FortéBio BioLayer interferometry.

In order to produce a spike-trimer protein with a high yield, stable prefusion structure, and the ability to self-trimerize via disulfide linkages, we designed and obtained the full length of the S-2p trimer protein extracellular domain and introduced mutations to abolish the S1/S2 cleavage by furin protease, which stabilizes the protein in a prefusion form (6, 30, Figure 2(a)). We fuzed the T4 trimerization domain to the amino acid residues 16–1209 of SARS-CoV-2 spike protein, and a histidine purification tag was added at the end. Then, the culture supernatant was collected 4 days after the transient transformation into 293F cells, during which a cell culture supplement was added every 24 hr. The recombinant S-trimer was successfully purified from serum-free cell culture supernatant, whose molecular weight was around 540–600 kDa, verified by gel filtration chromatography. The results of SDS–PAGE showed that the molecular mass of S-trimer after high glycosylation post-translational modifications was approximately 180 kDa under reducing conditions (Figure 2(b)). After SEC-HPLC, S-trimer had more than 96% purity (Figure 2(c)). When 2 *μ*g/ml (100 *μ*l/well) immobilized human ACE2 protein binds S-trimer, the EC_50_ of S-trimer is 8 ng/ml (Figure 2(d)). In addition, we determined the binding affinity of S-trimer to the human ACE2 using ForteBio BLI technology and MetaSPR sensor, respectively. The affinity value of the BLI (KD = 3.03 nM) (Figure 2(e)) was consistent with that of the MetaSPR sensor (KD = 1.19 nM) (Figure S1).

### 3.2. Assay Optimization

**Figure 3:**
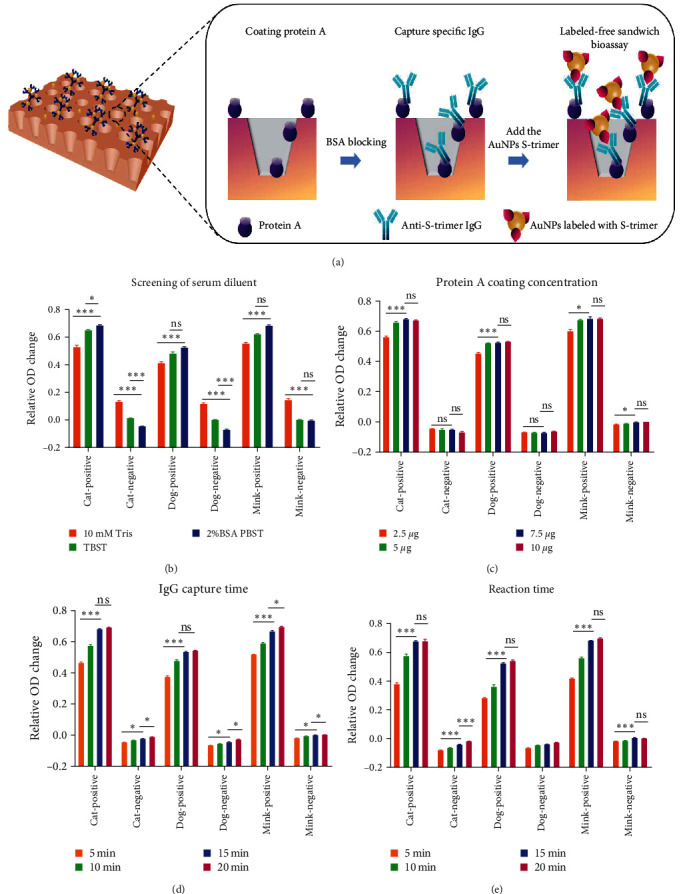
PEI-GNPs MetaSPR biosensor assay optimization: (a) procedure for the determination of S-trimer antibody using the MetaSPR biosensor; (b) PBST (pH7.45) base buffer supplemented with 2% (w/v) BSA was the best serum dilution buffer; (c) the maximum OD response in the sera of cats, dogs, and minks was obtained when the coating concentration of protein A was 7.5 *µ*g/ml; (d) the maximum OD response when capturing the serum IgG of cat, dog, and mink was obtained when 7.5 *µ*g/ml protein A was used for 15 min; (e) protein A, S-trimer antibody, and GNP-labeled S-trimer protein complex exhibited the maximum OD response after shaking at room temperature for 15 min. Data are shown as mean ± SD. Error bars represent the standard deviations from triplicates.

The modified protein A, which can bind the Fc portion of IgG antibodies in different species, was immobilized onto the surface of the PEI-GNPs MetaSPR biosensor in order to achieve further the goal of assessing multispecies sera of cats, dogs, minks, and other animals. After the total IgG antibody was captured by protein A on the surface of the biosensor, 100 *µ*l of GNP-labeled S-trimer protein at a concentration was added. During the reaction with continuous mild shaking at room temperature, protein A, anti-S-trimer IgG, and GNP-labeled S-trimer protein were combined to form a sandwich complex. Eventually, refractive changes at the surface of the biosensor after the formation of the sandwich complex are detected by a microplate reader and converted into a transmission light spectroscopy image (Figure 3(a)). We explored different serum dilutions, protein A coating concentrations, and antibody capture and reaction times to optimize performance. The maximum signal-to-noise ratio was obtained when PBST (pH 7.45) with 2% (w/v) BSA was used as serum diluent, significantly better than 10 mM Tris buffer (Figure 3(b)). In addition, the best detection in cat, dog, and mink sera was obtained when the coating concentration of protein A was 7.5 *μ*g/ml and the capture time of IgG was 15 min (Figures 3(c) and 3(d)). After five times PBST washing, when 100 *µ*l of GNP-labeled S-trimer protein with a concentration of 1 *μ*g/ml was added, the maximum response value could be reached by reacting with captured IgG for 15 min (Figure 3(e)).

### 3.3. Sensitivity and Reproducibility of SARS-CoV-2-Specific Binding Antibodies in Sera of Cats, Dogs, and Minks with PEI-GNPs MetaSPR Biosensor

**Figure 4:**
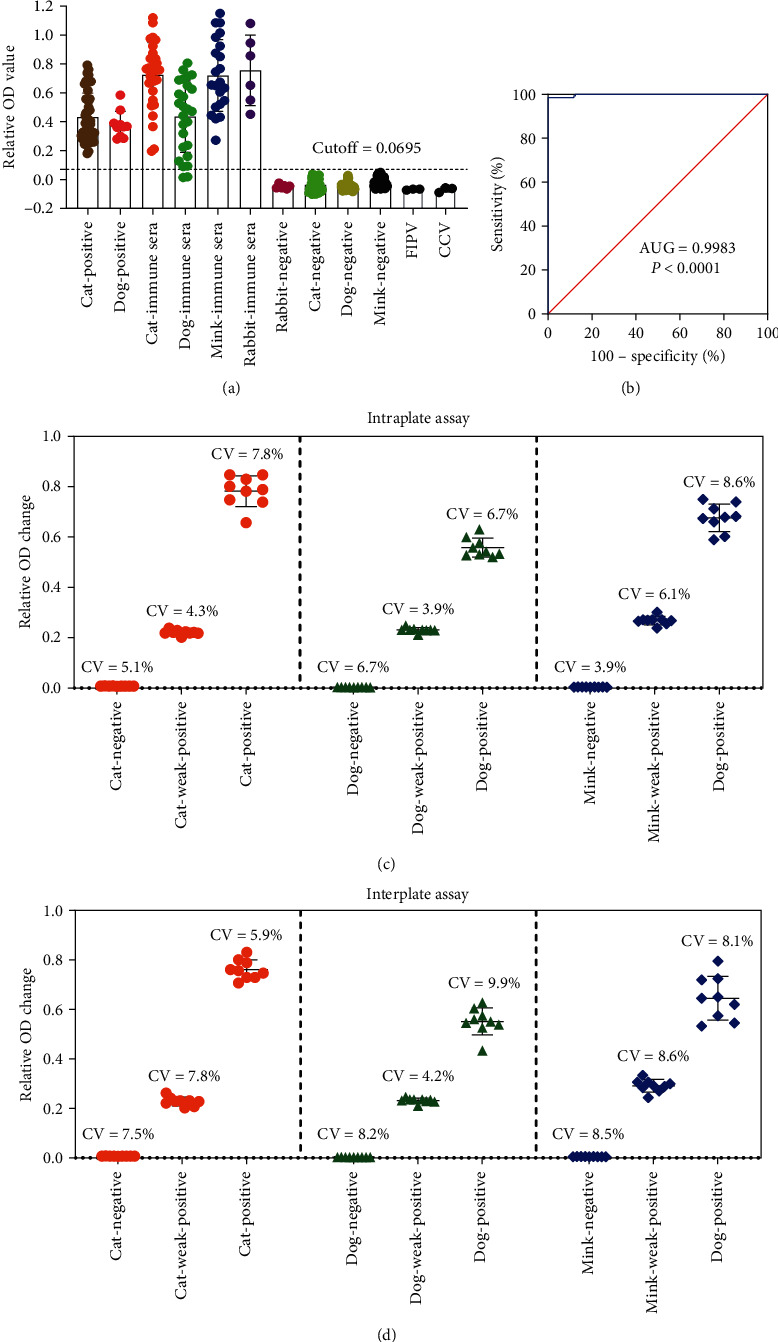
PEI-GNPs MetaSPR biosensor analytical performance: (a) detection of SARS-CoV-2 S-trimer-binding antibody via the PEI-GNPs MetaSPR biosensor assay from a panel of 315 serum samples; (b) ROC curve based on the obtained data; (c) nine intraplate replicates, including negative, weak, and strong positive sera from cats, dogs, and minks, analyzed in the PEI-GNPs MetaSPR biosensor; (d) nine interplate replicates, including negative, weak, and strong positive sera from cats, dogs, and minks, analyzed in the PEI-GNPs MetaSPR biosensor. Points represent individual samples.

The detection cutoff of the PEI-GNPs MetaSPR biosensor was determined with a receiver operating characteristic (ROC) curve using SARS-CoV-2-infected, vaccine-immunized, and control sera with known pseudovirus neutralization titers. The vaccine-immunized serum contains strongly/weakly positive samples with a clear background since it was obtained following a single or double dose of immunization. As shown in Figure 4(a), S-trimer binding antibodies were detected in convalescent sera of dogs and cats and immune sera of dogs, cats, and minks. Conversely, they were not in sera of naïve dogs, cats, and minks collected before the outbreak. In addition, the sera positive for feline infectious peritonitis virus and canine coronavirus did not cross-react with S-trimer. According to the corresponding ROC curve, there is a good cluster between negative and positive sera when the diagnostic cutoff value is assigned to 0.0695 (*P*  < 0.0001). The overall concordance between the results of the PEI-GNPs MetaSPR biosensor detection system and pVNT was 99.36% (313/315) (Figure 4(b)). The two discrepant samples were collected from dog sera after 1 week of single-dose immunization; the pVNT result was positive, and the neutralization titers were 1 : 20 (inhibition of 67.80% and 58.64%). Therefore, our system shows good detection specificity.

Next, to evaluate the reproducibility of the system, the negative, weak, and strongly positive dogs, cats, and minks sera were selected for 9-times repeated assays intra- and interplate. The results demonstrated that the intra- and interplate repeatability of the PEI-GNPs MetaSPR biosensor was lower than 10% (coefficient of variation) and was not affected at the species or antibody levels, which is within the acceptable range (CV < 15%) (Figures 4(c) and 4(d)).

**Figure 5:**
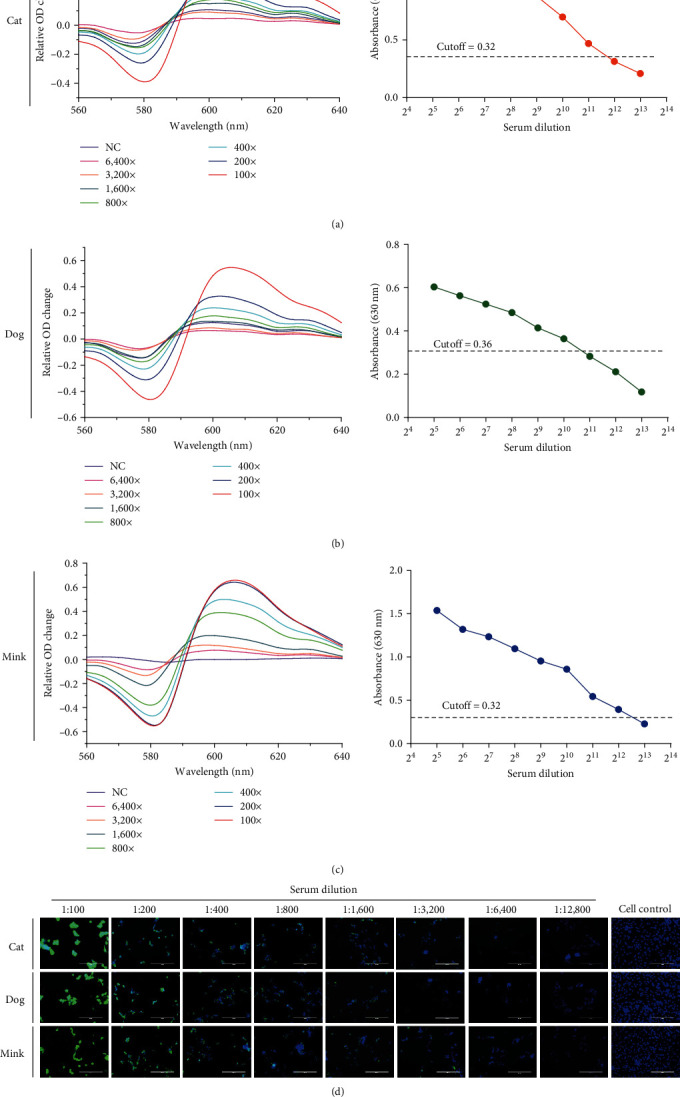
Sensitivity comparison between the PEI-GNPs MetaSPR biosensor and current methods: (a) comparison of detection sensitivity between the PEI-GNPs MetaSPR biosensor system and S-trimer-ELISA in cat serum; (b) comparison of detection sensitivity between the PEI-GNPs MetaSPR biosensor system and S-trimer-ELISA in dog serum; (c) comparison of detection sensitivity between the PEI-GNPs MetaSPR biosensor system and S-trimer-ELISA in mink serum; (d) comparison of detection sensitivity between the PEI-GNPs MetaSPR biosensor system and indirect immunofluorescence using SARS-CoV-2 antibody detection. The cat, dog, and mink SARS-CoV-2-positive sera were diluted from 1 : 100 to 1 : 16,000-fold. All of the above results are representatives of at least three independent experiments.

Finally, to determine the sensitivity of the system, samples of dogs and cats convalescent sera and minks inactive vaccine immune sera were selected as standard and compared to the S-trimer-ELISA assay in terms of sensitivity. We obtained transmission light spectroscopy imaging fitting curves of the standard sera within the 1 : 100–1 : 6,400 linear dilution range. Also, the curve of the negative serum control group showed a very small change. The standard sera of cats, dogs, and minks can obtain positive results even after being diluted up to 3,000, 1,600, and 6,400 times, respectively, with the PEI-GNPs MetaSPR biosensor detection assay. Nonetheless, sensitivity was significantly higher than in S-trimer-ELISA (1,024, 2,048, and 4,096 times for cat, dog, and mink sera, respectively, Figure 5(a)–5(c)). Most importantly, similar to that in the ELISA method, the relative OD change in this detection system is directly proportional to the concentration of binding antibodies in the serum. Additionally, in cats, dogs, and minks, the PEI-GNPs MetaSPR biosensor detection method shows similar detection sensitivity to the conventional SARS-CoV-2 indirect immunofluorescence assay (Figure 5(d)).

## 4. Discussion

There have been multiple reports of animal infections since the global COVID-19 pandemic started. As a result, techniques for screening cases of animal infection have been developed; however, the majority of these techniques have drawbacks, including being species-dependent, labor-intensive, and having poor throughput [24, 28]. Here, we have developed the PEI-GNPs MetaSPR biosensor detection system. The components of the test kit and the detection stages (incubation, washing, and capture) make up this detection system (MetaSPR biosensor preincubated with protein A, the GNP-labeled SARS-CoV-2 S-trimer antigen). The specific operational steps are as follows: the serum to be detected is diluted at a ratio of 1 : 100 and applied to the MetaSPR biosensor pre-coated with 7.5 mg/ml of protein A. After 15 min, the total IgG is captured, followed by washes with PBST five times. The initial OD value of the biosensor is then read. Subsequently, 100 *µ*l of GNP-labeled S-trimer protein at a concentration of 1 *μ*g/ml is added.

The final OD value is read following a 15-min reaction, and the preprogramed software computes and interprets the relative OD values. When used as a targeted, species-independent technique in extensive animal seroepidemiology screening investigations, this assay may prove to be highly valuable. Different from the traditional ELISA method, which uses an enzyme-labeled plate, this approach is based on an unlabeled MetaSPR biosensor with an extraordinary optical transmission effect integrated into a standard 96-well plate. The biosensor has the potential to generate an extremely sensitive response to minute variations in the local refractive index resulting from the antigen–antibody sandwich. The PEI-GNPs significantly improved this sensitivity. The detection sensitivity in this assay is significantly higher than that of the traditional ELISA, and it is comparable to the gold-standard indirect immunofluorescence method. In order to evaluate the biosensor, 315 sera with distinct negative and positive backgrounds were used, and the results showed a high compliance rate of up to 99.36% (313/315). The inconsistent results of one sample may be due to antibody degradation (neutralization titer was 1 : 20) after freezing and thawing. Subsequent loss efficacy sample pVNT confirmed this.

**Figure 6:**
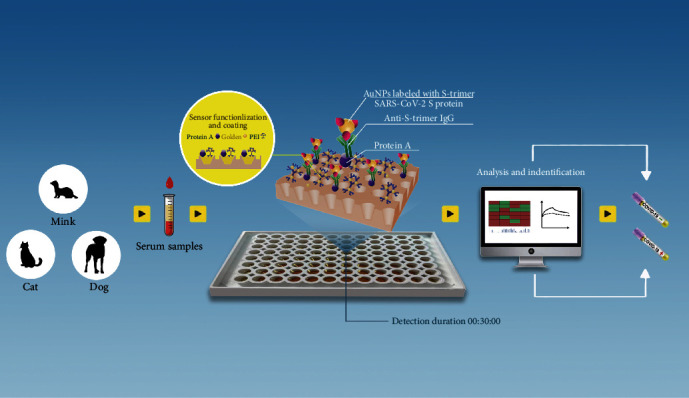
Diagram showing the multispecies PEI-GNPs MetaSPR biosensor SARS-CoV-2 antibody detection.

Furthermore, the PEI-GNPs MetaSPR biosensor system significantly reduces detection time and streamlines the detection process. Specifically, incubation and repeated washing steps are simplified and replaced by three steps: antibody capture, washing, and reaction. Complex, error-prone steps are replaced by automatic analysis with preprogramed software, where the total time from sample loading to result in identification is only 30 min (Figure 6). Thus, this detection system is more appropriate for broad serological screening than traditional methods because it has simple operation, quick assay time, high throughput, good sensitivity, and a high degree of automation compared to other serological detection methods.

The protein A preimmobilized at the bottom of the biosensor is the basis of the PEI-GNPs MetaSPR biosensor detection system, which enables multispecies detection without the need to reoptimize assays using the appropriate species-specific anti-IgG conjugate. Total IgG is captured in the sera of cats, dogs, and minks. Therefore, this system targets a wider range of species and is less restricted by experimental materials, while it can also effectively reduce labor intensity and reduce total detection time when processing a large number of samples of different species. The quality of the samples, which is determined by the source, storage circumstances, and duration of storage, is intimately linked to the quality of the test results. The detection system uses protein A to enrich and purify the IgG for washing, effectively avoiding substances that can interfere with the detection results. Bilirubin, complement, hemoglobin, IgA, and IgM, as well as foreign components like bacterial infection and anticoagulants, resulting in a 100% specificity for this system.

According to a report, targets such as S1 and the RBD were utilized to build serological diagnostic techniques, which also showed good performance [28, 40, 41]. Because the full-length spike protein contains a higher number of epitopes [27, 42], in this study, full-length S protein was used as a candidate diagnostic target, and recombinant full-length S expressed in HEK293 cells. Mutations in furin protease (R683A, R685A) and S-2P (K986P, V987P) produced the stabilized protein in a prefusion form. N terminal fusion to T4 Trimer-Tag allows the soluble spike protein to form a disulfide bond-linked homotrimer [43]. The highly glycosylated S-trimer exposes key epitopes of antigen by folding correctly, allowing them to exhibit a higher affinity to the ACE2-HFc receptor than other studies [44]. Additionally, S-trimer protein was linked with GNP-labeled material to improve preservation stability and the detection signal, which would make S-trimer stable in liquid solution formulations at 4°C for at least 6 months (result not shown). As a result, the detecting system exhibits strong repeatability.

This study does, however, have certain drawbacks. Once the technique is accessible, it will be crucial for understanding COVID-19 epidemiology and identifying the animals that do not exhibit any symptoms. Still, they are unlikely to play any role in screening or diagnosing early infections. Nonetheless, serology may be useful for confirming the diagnosis of COVID-19. Our system can be applied to the antibody detection of cats, dogs, and mink, which are the largest groups with SARS-CoV-2 spillover cases. However, its applicability to multiple species is affected by the strength of the binding force between protein A and the lgG Fc segment.

Additionally, more research is required to determine whether this approach may be applied to certain vulnerable uncommon wild species in which the IgG Fc region binds to protein A only weakly. At present, the Omicron strain is the most prominent, and there have been reports of infected cases in cats, dogs, and minks [46]. Also, the antigenic properties of the Omicron strain have changed greatly. Despite research indicating that sera immunized against several mutant strains can be identified using wild-type S protein, no assessment was carried out because there were insufficient clinical serum samples [47].

In conclusion, we developed a multispecies (cat, dog, mink) SARS-CoV-2 antibody detection system with short operating time, high sensitivity, and a high degree of automation based on the PEI-GNPs functionalized MetaSPR biosensor and stable, high affinity, prefusion conformation S-trimer. This system provides an effective means for epidemiological screening in companion animals and minks and identification of asymptomatic infection. In order to achieve greater host applicability, we will now investigate the applicability of this approach in additional species.

## 5. Conclusion

In this study, we developed a multispecies SARS-CoV-2 antibody detection method based on the PEI-GNP MetaSPR biosensor system and a high-affinity prefusion conformation S-trimer. This method exhibits a short operating time, high sensitivity, and high level of automation, allowing for detection within 30 min with 100% specificity for cats, dogs, and minks. Additionally, the detection of pseudovirus in clinical and immune sera showed a high coincidence rate of 99.36% (313/315). Therefore, this method stands as an efficient approach for conducting COVID-19 epidemiological screening in animal serum.

## Data Availability

Data sharing is not applicable to this article as no datasets were generated or analyzed during the current study.

## Supplementary Materials

Supplementary Materials Figure S1: determination of the binding affinity between S-trimer and human ACE2-HFc receptor by MetaSPR sensor.
