# Supplementary material for: Rapid, Multispecies Detection of SARS-CoV-2 Antibodies via a Meta-Surface Plasmon Resonance Biosensor
Source: Transbound Emerg Dis. 2024 Jan 19;2024:9350822. doi: 10.1155/2024/9350822 (PMC12016715; doi:10.1155/2024/9350822)
Supplement: Supplementary Materials — Figure S1: determination of the binding affinity between S-trimer and human ACE2-HFc receptor by MetaSPR sensor. [file 9350822.f1.docx]

**Figure S1: Determination of the binding affinity between S-trimer and human ACE2-HFc receptor by MetaSPR sensor.**
